# Seismic Imaging of Thickened Lithosphere Resulting From Plume Pulsing Beneath Iceland

**DOI:** 10.1029/2018GC007501

**Published:** 2018-06-22

**Authors:** Catherine A. Rychert, Nicholas Harmon, John J. Armitage

**Affiliations:** ^1^ National Oceanography Centre Southampton, Ocean and Earth Sciences University of Southampton Southampton UK; ^2^ Dynamique des Fluides Géologiques, Institut de Physique du Globe de Paris Paris France

**Keywords:** Iceland, scattered wave imaging, geodynamic modeling, lithosphere, asthenosphere

## Abstract

Ocean plates conductively cool and subside with seafloor age. Plate thickening with age is also predicted, and hot spots may cause thinning. However, both are debated and depend on the way the plate is defined. Determining the thickness of the plates along with the process that governs it has proven challenging. We use S‐to‐P (Sp) receiver functions to image a strong, persistent LAB beneath Iceland where the mid‐Atlantic Ridge interacts with a plume with hypothesized pulsating thermal anomaly. The plate is thickest, up to 84 ± 6 km, beneath lithosphere formed during times of hypothesized hotter plume temperatures and as thin as 61 ± 6 km beneath regions formed during colder intervals. We performed geodynamic modeling to show that these plate thicknesses are inconsistent with a thermal lithosphere. Instead, periods of increased plume temperatures likely increased the melting depth, causing deeper depletion and dehydration, and creating a thicker plate. This suggests plate thickness is dictated by the conditions of plate formation.

## Key Points

- S‐to‐P receiver functions image laterally variable lithospheric thickness beneath Iceland
- Lithospheric thickness is greatest in regions formed during hypothesized periods of greater plume flux or temperature
- Geodynamic and waveform modeling suggest melt beneath the plate, with thickness dictated by the conditions of formation

## 1 Introduction

The lithosphere‐asthenosphere boundary (LAB) represents the base of the tectonic plate, a fundamental boundary in plate tectonics. It represents the transition of the rigid plate to the weaker asthenosphere. Since it is well established that temperature plays a large role in the strength of materials, the LAB is typically assumed to follow an isotherm (Parsons & Sclater, 1977), with ocean plates progressively thickening as they cool and age (Parsons & Sclater, 1977) and thinning in the presence of hot spots (Detrick & Crough, 1978). In this model, seismic velocities are predicted to gradually decrease from the lithosphere to the asthenosphere (Jackson & Faul, 2010; Rychert et al., 2010; Tharimena et al., 2017; Rychert & Harmon, 2018). However, several observations of sharp velocity gradients (Kawakatsu et al., 2009; Rychert et al., 2005; Rychert & Shearer, 2009) and discontinuities at constant depth beneath the oceans (Gaherty et al., 1999; Tan & Helmberger, 2007; Tharimena et al., 2017) suggest a chemical boundary or a variation in melt might exist that could affect the strength of the mantle (Hirth & Kohlstedt, 1995; Jackson et al., 2006) and therefore influence the location of the LAB (Rychert et al., 2005). A compositional boundary might reflect the depth extent of depletion caused by melting during plate formation or subsequent melting due to hot spot anomalies (Gaherty et al., 1999; Hall & Kincaid, 2003; Hirth & Kohlstedt, 1996; Jordan, 1978). Melt ponding, possibly controlled by the solidus (Sparks & Parmentier, 1991) or the melt‐solid density contrast, might also determine the depth of the LAB (Sakamaki et al., 2013). However, the exact relationship between composition and/or melt variations and plate thickness has yet to be established.

Iceland is a unique opportunity to study the definition of the plate given its location above a thermal plume anomaly currently interacting with a mid‐ocean ridge. Mid‐ocean ridges are ideal locations to study the plate definition, given the simple and short history of the plate. Ocean islands like Iceland provide rare in situ constraints on the oceanic lithosphere, given the difficulties of ocean bottom seismic experiments. Hypothesized temporal variations in the magnitude of the thermal plume anomaly beneath Iceland may also be evaluated (Ito, 2001; Jones et al., 2002). Here we use Sp receiver functions to investigate discontinuity structure related to the LAB beneath Iceland. We also perform numerical modeling of mantle flow, temperature and melting beneath Iceland, and translate the thermal structure to predicted seismic velocity and receiver functions to test whether a purely thermally defined lithospheric plate may explain our observations, or another mechanism is required.

## 2 Methods

### 2.1 Data Processing

We consider 4,265 waveforms recorded by stations in the IRIS database (http://www.iris.edu) from 1996 to 2005 and epicentral distances 55–80°. We hand‐pick the data, selecting the best 618 *S*‐waves. We calculate receiver functions using two methods of Sp deconvolution, a multitaper (Helffrich, 2006; Rychert et al., 2012) method which is stable for individual waveforms and provides good 3‐D structure and a simultaneous deconvolution (Rychert et al., 2007) which can verify the robustness of results. We experiment with a variety of frequency bands. A more limited band, typical for ocean islands (Li et al., 2004; Rychert et al., 2013), gives simpler waveforms, which we use for waveform modeling 0.05–0.14 Hz. The interpreted features of the stacked 3‐D image are robust regardless of filtering and we present a wider band, 0.03–0.25 Hz, to demonstrate the highest resolution we could achieve. Deconvolved waveforms are multiplied by negative one, to match polarity of Ps receiver functions.

In the multitaper deconvolution, we eliminate individual unstable deconvolutions, characterized by ringing, where large amplitudes recur periodically for Sp delay times of 0 to −70 s (Rychert et al., 2012, 2007). This leaves 304 waveforms for the final multitaper imaging. The receiver functions are migrated to depth in 3‐D. Migration model crustal thicknesses correspond to the value from the surface wave model (Li & Detrick, 2006) at the Sp conversion points at 30 km depth. The crustal shear velocity beneath the station from surface waves is assumed. Crustal Vp/Vs is assumed to be 1.75, between the median (1.74) and the mean (1.76) values for the island based on Ps and PsPs arrival times (Kumar et al., 2007), and within the range from several active source seismic studies (1.72–1.79) (Staples et al., 1997; Tryggvason & Bath, 1961; Weir et al., 2001). In the mantle, we assume a 1‐D shear velocity structure from surface waves, and Vp = 8.0 km/s. Multitaper waveforms are binned at 1 km depth spacing on a 0.25° by 0.25° grid from 0 to 80 km depth and a 0.75° by 0.75° grid at deeper depths. The grid is smoothed with a radius corresponding to the Fresnel zone of the waveform. Only bins with greater than 3 waveforms where data amplitude exceeds the formal error bar (2 standard deviations from the mean) are included.

For the simultaneous deconvolution we divide the data into two large bins, one in the southwest (305 waves) and one in the northeast (313 waves). Large bins are necessary to attain a robust result with this method. Migration model parameters correspond to the 1‐D average of the migration model described above, based on surface waves (Li & Detrick, 2006).

### 2.2 Synthetic Waveform Modeling

We forward model the multitaper and simultaneous deconvolution results assuming a 1‐D model and using a propagator matrix method to generate synthetic seismograms (Keith & Crampin, 1977). The synthetics are processed and deconvolved as receiver functions using the same parameters as the data (Helffrich, 2006; Rychert et al., 2012). The migration model shear velocities are updated for consistency with the forward modeling. We assume the crust is a single layer when forward modeling, for simplicity. The dominant period of the waveforms is assumed to be 12 s and is determined by auto‐convolution and considering the character of deconvolved waveforms.

Error bars on the simultaneous deconvolution waveforms represent 95% confidence as determined by a bootstrap with 100 repeats. Error bars on the multitaper deconvolutions represent the formal 95% confidence limits. Errors in the magnitude and sharpness of the velocity contrast are determined by changing the forward model to reach the limits of the bootstrap error bar. Error in depth to the discontinuities (±5 km for the Moho and ±6 km for the LAB) reflects variability caused by changing the migration model Vp/Vs assumption by 5%, leaving shear velocity fixed.

In filtered versions of the data (0.05–0.14 Hz), the Moho appears as a broad phase, likely owing to interference between the Moho at 25 and 40 km and the phase from the base of the extrusives at 8–10 km. We do not model this complexity. Testing indicates that complex crustal structure does not greatly influence our results from deeper phases. Changing the crustal shear velocity by 10% affects the estimated magnitude of the LAB phase by <1%. We also test the effect of assuming a different crustal thickness model (Kumar et al., 2007) for migration, but find it affected results from the LAB discontinuity by <2 km depth.

### 2.3 Geodynamic Modeling

We solve Stokes flow with the Boussinesq approximation (Armitage et al., 2008; Moresi et al., 1996; Nielsen & Hopper, 2004). We assume that the mantle deforms by dislocation creep (Levy & Jaupart, 2011), that the presence of partial melt leads to a weakening that has an exponential relationship between bulk viscosity and porosity (Kelemen et al., 1997), and that the dehydration of the solid matrix leads to a strengthening of the upper mantle (Hirth & Kohlstedt, 1996).

This leads to the following rheological law:

$$\eta =\chi_{\mathrm{dehy}}e^{-a\phi }A^{\frac{1}{n}}\;\text{exp}\left(\frac{E+pV}{nRT}\right){\dot{I}}^{(1-n)/n}$$

where the various constants are given in Table 1. The parameter χ_dehy_ is equal to 1 until 2% melt is generated, where it increases to 10. This is to simulate the strengthening effect of the removal of volatile compounds on the solid mantle matrix. We choose to increase the strength by only one order of magnitude given the on‐going debate for actual effect of water on the strength of mantle rocks (Fei et al., 2013). The Stokes solver applies a viscosity cut‐off to avoid very large differences in viscosity within the numerical domain. The maximum viscosity is 10^23^ Pa s, and the minimum is 10^17^ Pa s.

**Table 1:** Geodynamic Model Parameters

Parameter	Value	Units	Reference
a	45		Kelemen et al. ( 1997)
A	8.64 × 10^−12^		
E	523	kJ mol^−1^	Korenaga and Karato ( 2008)
n	3.6		Korenaga and Karato ( 2008)
V	4	cm^3^ mol^−1^	Korenaga and Karato ( 2008)

We present a model of extension of the upper mantle, where the extension is driven by a divergent kinematic velocity of 10 mm/yr. The velocity is applied at 20 km depth, to approximate the effect of an increased crustal thickness on the lithosphere thickness. The velocity boundary conditions are of free slip and for temperature they are fixed at the top, 0°C, and bottom, 1,450°C, and are of zero gradient at the sides. This is the model we present here, although we also tested models assuming potential temperatures from 1,400°C to1,600°C in 50°C increments. To drive extension at 20 km depth requires a high model resolution, 2.7 × 0.7 km (1,024 × 1,024 elements), so that the velocity can be applied at the nodal points within the model domain. The center of extension is moved laterally and the resultant temperature and melt production is plotted after 6.2 My postmigration. This is to simulate the ridge jump at ∼6–7 Ma.

For both models where the velocity is applied at the surface and at 20 km depth we find that the temperature field is very similar, despite the change in the depth of the driving velocity condition. This is because the flow field within the upper 50 km is reasonably similar. The vertical profile of the model viscosity shows an increase in viscosity that is due to the reduction in temperature. Above a depth of 30 km, the predicted mantle flow is sufficiently slow that thermal diffusion dominates over advection, and the isotherms are controlled by heat conduction. In the models viscosity increases to 10^23^ Pa s, which is the cut‐off. For the model where the divergent velocity is applied on the surface this viscosity increase to the cut‐off is gradual. However, when the velocity is applied within the model domain, we create a region of reduced viscosity where the flow is imposed, as the rheology is a function of strain rate and viscosity reduces with increasing strain rates. Yet this difference in viscosity is too minor to alter the overall flow of the mantle and therefore significantly alter the thermal structure of the model lithosphere‐asthenosphere system.

We convert the thermal structure to seismic velocities (Jackson & Faul, 2010), assuming a primarily olivine mantle. The method accounts for attenuation effects based on laboratory scalings and we assume a grain size of 20 mm. The seismic velocity structure is calculated for each element in the model. We calculate the predicted receiver functions from the seismic velocities using a 1‐D reflectivity code (Shearer & Orcutt, 1987) assuming a 20 km thick crust in averaging the velocity structure over 50 km wide bins. We use the same frequency band used in our study 0.03–0.25 Hz.

## 3 Results

**Figure 1:**
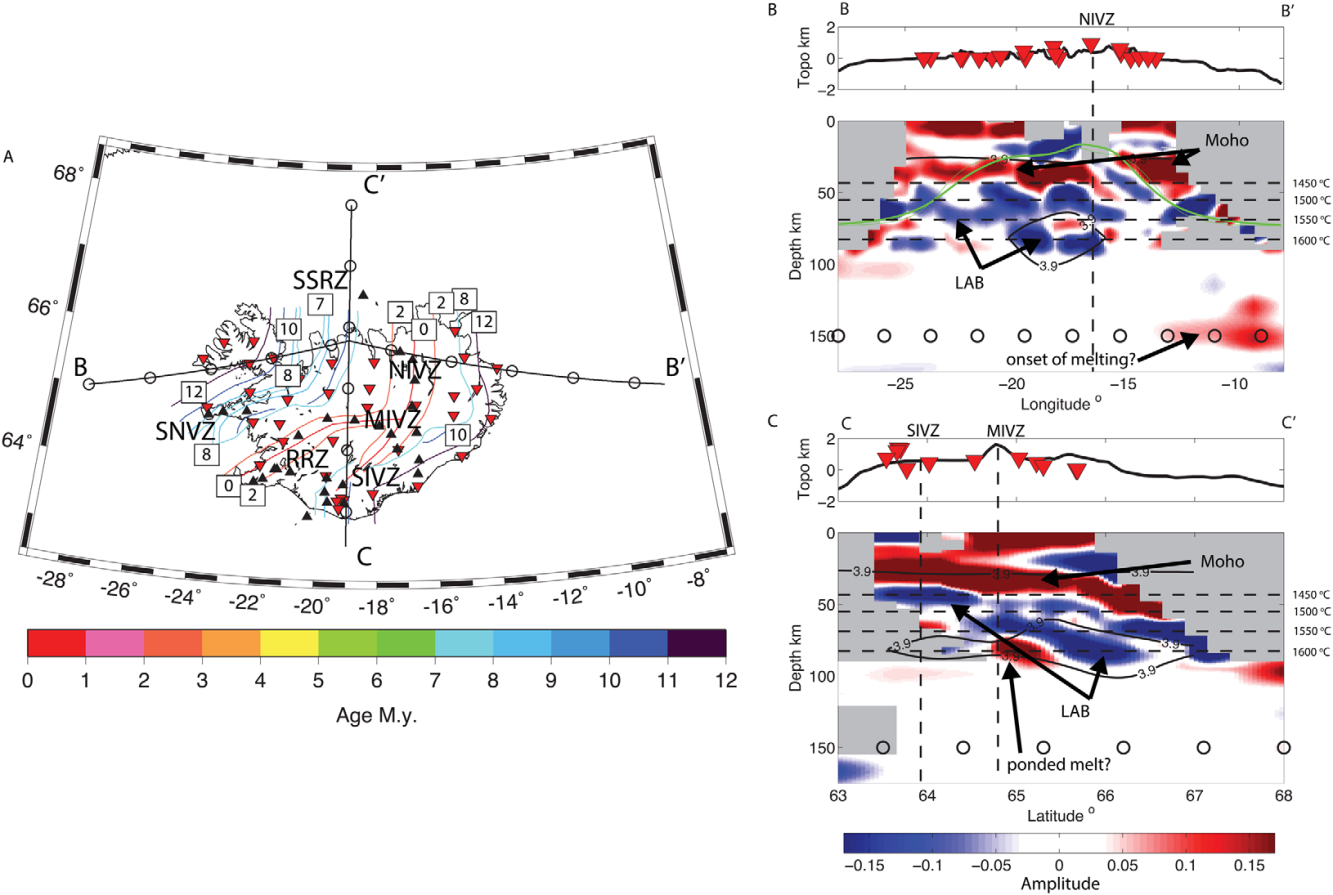
Map of the study region and cross sections through the migrated Sp multitaper receiver function model. (a) Color contours show lithospheric age. Iceland coast outlined in black. Some of the active volcanic zones are labeled with abbreviations as follows: SNVZ, Snaefellsnes Volcanic Zone; MIVZ, Mid‐Iceland Volcanic Zone; SIVZ, South Iceland Volcanic Zone; RRZ, Reykjanes Rift Zone; NIRZ, North Iceland Rift Zone. Extinct volcanic zones: SSRZ, Snæfellsnes‐Skagi Rift Zone. Holocene volcanos are indicated with black triangles (Venzke, 2013). (b, c) Cross sections through the migrated Sp multitaper receiver function model. Colors indicate the polarity of seismic discontinuities from receiver functions, positive, velocity increase with depth (red) and negative, velocity decrease with depth (blue). Open circles at 150 km depth are spaced at 100 km and plotted as reference points. Topography is plotted at top, with exaggeration. Regions with insufficient data are shaded grey. Inverted red triangles show seismic station locations. Black contours show shear wave velocity from surface waves (Li & Burke, 2006), as labeled. Green line shows the 1,000°C isotherm from the geodynamic model. Horizontal dashed black lines indicate 20% depletion for a range of mantle potential temperatures, as labeled. Error in depth of the LAB phase is ∼6 km, as discussed in section 3.

**Figure 2:**
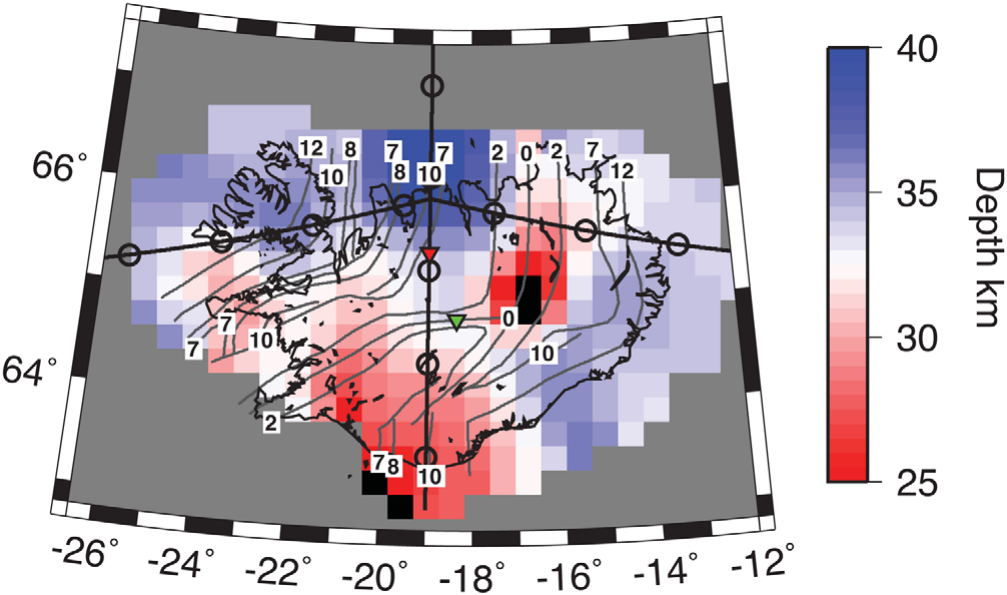
Depth to the Moho from the multitaper method, map view. Bins with fewer than 3 waveforms are shaded grey. Black lines outline Iceland coast. Dark grey lines show age contours: 0, 2, 7, 8, and 12 My, as labeled. Cross section lines of Figure 1 are drawn as a thick black horizontal and vertical lines. Red and green inverted triangles show waveform modeling locations (Figure 4). Error in depth of the Moho is ∼5 km, as discussed in section 3. Black shading shows the area where the Moho phase at similar depths (25–40 km) is not detected. Depths are determined automatically from migrated receiver function model.

Sp receiver functions image a shallow positive phase at 3–9 km depth (Figure 1). We also image a deeper positive phase that likely represents the Moho at 25–40 ± 5 km depth (Figures 1 and 2). The two phases are pervasive across the region except at the intersection of the rift segments in the center of the island where we image a single positive discontinuity at 14 ± 5 km depth (area of black shading, Figure 2).

**Figure 3:**
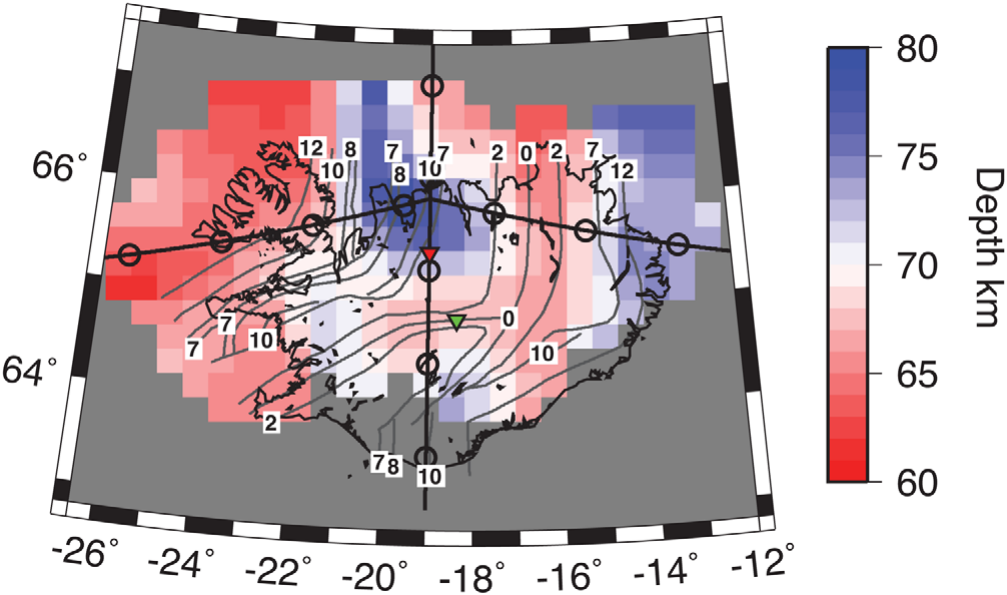
Depth to the negative discontinuity (LAB) from the multitaper method, map view. Bins with fewer than 3 waveforms or amplitudes less than 0.05 are shaded grey. Black lines outline Iceland coast. Dark grey lines show age contours: 0, 2, 7, 8, and 12 My as labeled. Cross section lines of Figure 1 are drawn as thick black horizontal and vertical lines. Red and green inverted triangles show waveform modeling locations (Figure 4). Error in depth of the LAB phase is ∼6 km, as discussed in section 3. Depths are determined automatically from migrated receiver function model.

**Figure 4:**
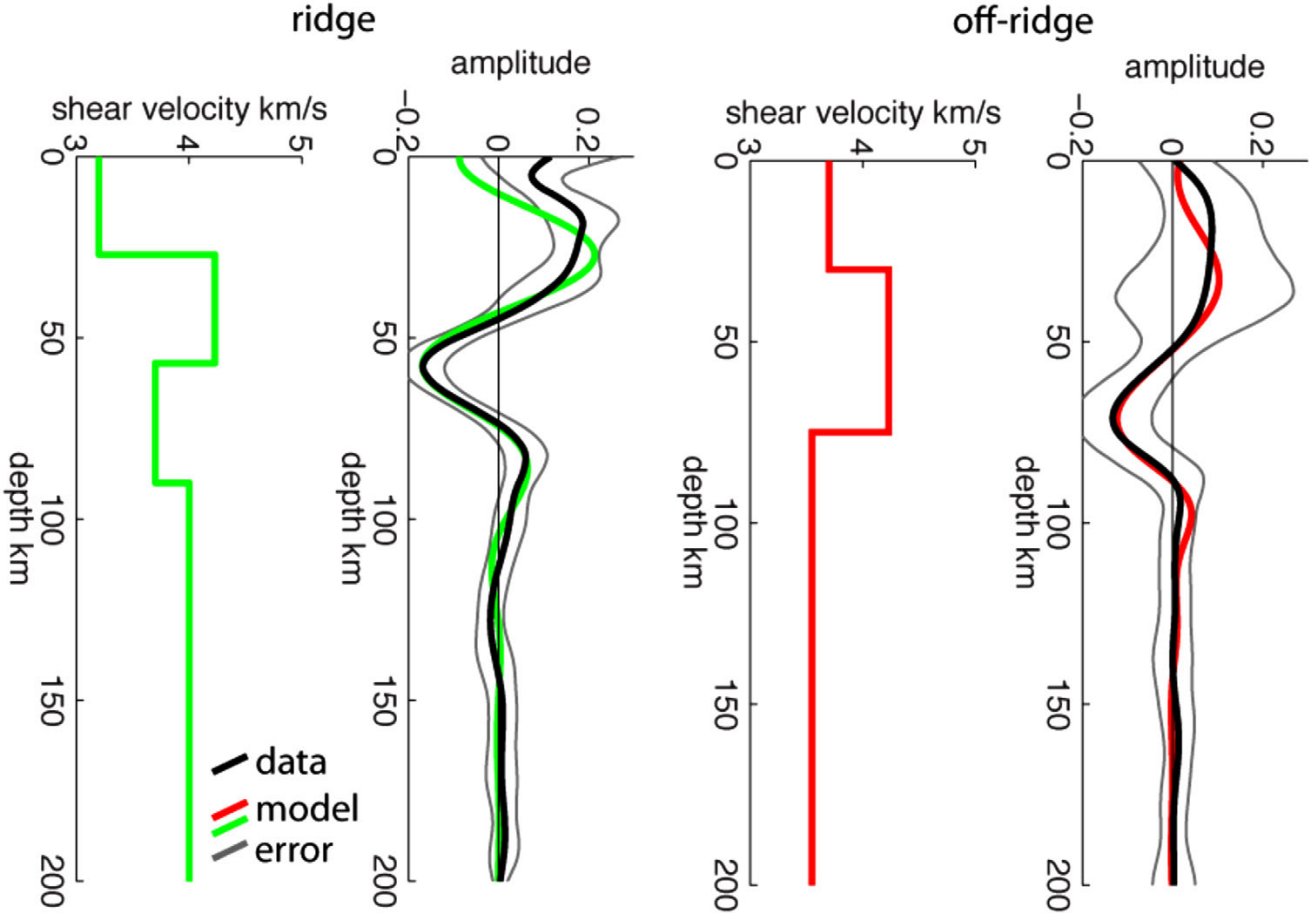
Waveform modeling of Sp 3‐D multitaper model (black) beneath the ridge and beneath the 9 My age contour (locations of triangles in Figure 3) are compared to synthetics from forward modeling (red and green lines). Corresponding shear velocity profiles are shown to the left of the data/synthetics plots. Error bars indicating 95% confidence from bootstrap are shown in grey.

We image a negative phase, velocity decrease with depth at 61–84 ± 6 km depth in the multitaper deconvolution (Figures 1 and 3). Beneath the ridge axis the phase is shallow, 60–70 km (Figure 3). The phase is imaged deeper just outside the ridge centered Quaternary volcanic area, with the deepest realizations approximately centered on the 9 Ma isochron (Figure 3). The receiver function1‐D profile in this region has two negative phases (Figure 1b), although the deeper phase is the most robust, interpretable feature, getting more pronounced when filtered to longer period. The phase is then shallower, 60–68 km, beneath oldest aged lithosphere in the northwest. The depth variations are independent of those of the Moho (Figures 2 and 3). The phase represents a strong velocity decrease with depth, 15 ± 7% beneath a 9 My old plate and 10 ± 5% beneath the South Iceland Volcanic Zone (SIVZ), as suggested by synthetic waveform modeling (Figure 4). The phase may be associated with the lithosphere‐asthenosphere boundary, which will be assessed in subsequent sections.

A positive phase, velocity increase in depth is imaged sporadically in the multitaper cross sections at 80–90 km depth. The phase appears in the locations of the slowest seismic velocity anomalies from surface waves (Figure 1). It is typically imaged at the deeper limit of the 3.9 km/s contour where negative LAB phase is also shallow (Figure 1c).

**Figure 5:**
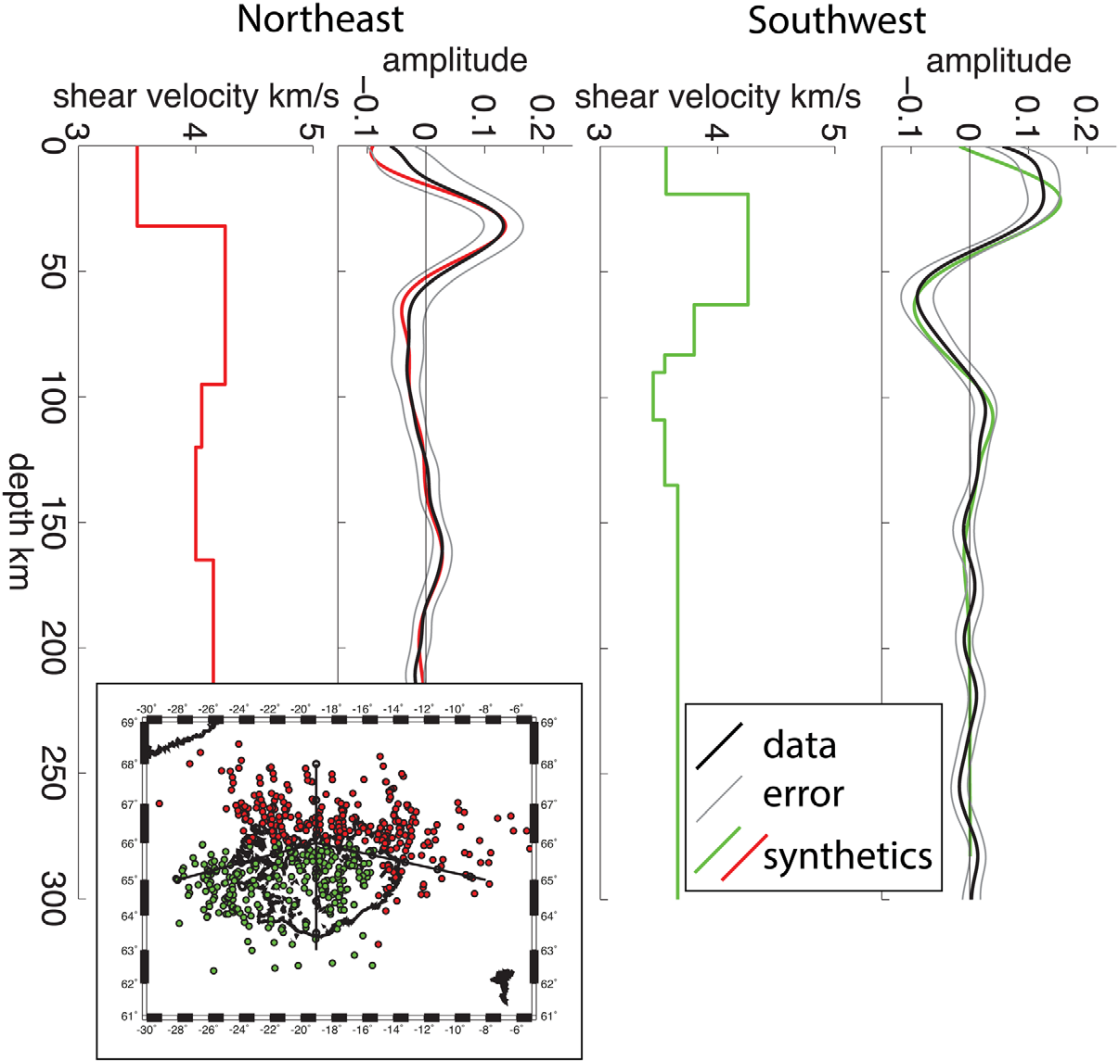
Waveform modeling of Sp data from the simultaneous deconvolution. Sp data (black) binned by conversion point bins at 100 km depth into 2 bins (red and green dots in inset map) are compared to synthetics from forward modeling for the southwest (green) and northeast (red). Corresponding shear velocity profiles are shown to the left of the data/synthetics plots. Error bars indicating 95% confidence from bootstrap are shown in grey.

**Figure 6:**
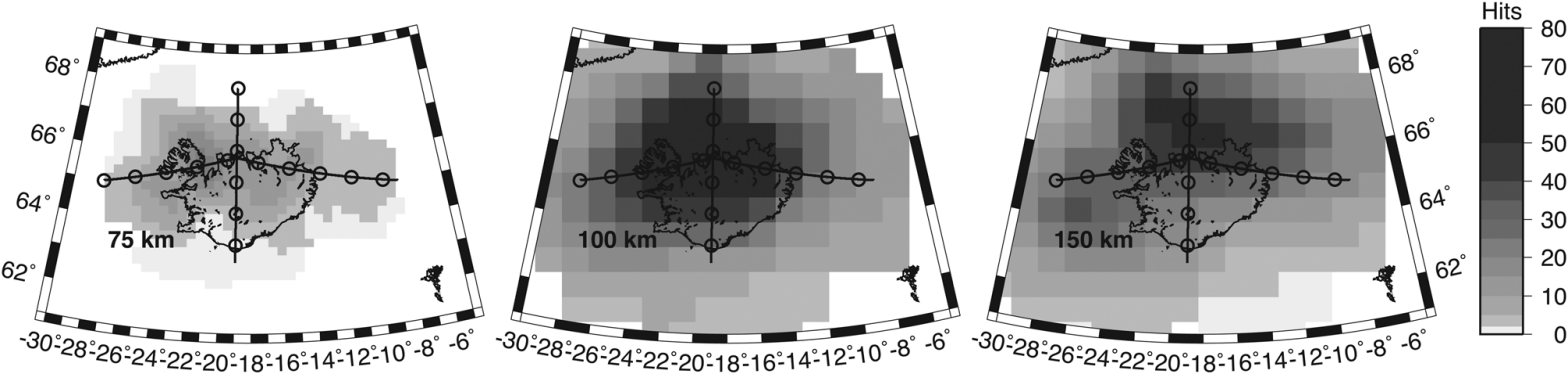
Hit count map for the multitaper method at 75 (left), 100 (middle), and 150 (right) km depth. The number of waveforms averaged in each bin is shown by grey scale, 0 (white) to up to 80 (black). Coastlines are outlined in black. Black line indicates cross section location.

A positive phase, velocity increase with depth is also imaged at 150 km in both the large northeastern simultaneous deconvolution bin (Figure 5) and east of the island in the 3‐D multitaper model (Figure 1). Modeling the simultaneous deconvolution suggests a 4 ± 1% velocity increase (Figure 5), although the data included averages over a large lateral area and therefore may underpredict the magnitude. Modeling the phase from the multitaper deconvolution is challenging given that it is at the edge of the model, and structure directly above it is not well‐resolved (Figures 1 and 6). We model the phase assuming a shallow discontinuity structure from the area adjacent to the discontinuity and find a large ∼17%, velocity increase. Therefore, the discontinuity likely represents a 4–17% velocity increase with depth. The forward modeling suggests that the velocity discontinuities could be sharp or occur over up to ∼20 km depth without changing the shape of the synthetic receiver function or as wide as 50 km depth before reaching the bounds of the error bars from bootstrap.

**Figure 7:**
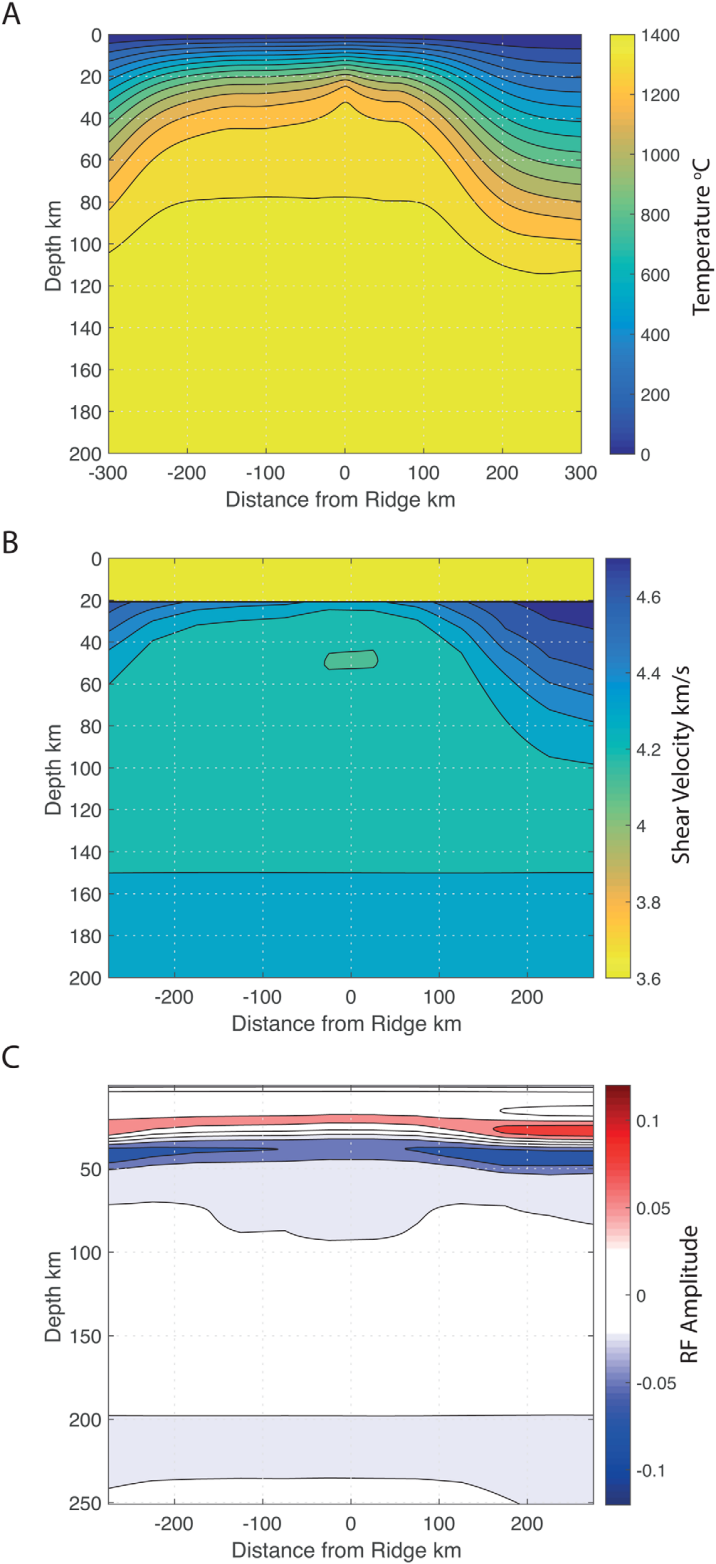
Geodynamic model of extension for the upper mantle. (a) Temperature, (b) seismic shear wave velocity, and (c) receiver function predictions are shown. The model shows results 6.2 Myr after a 100 km lateral shift in extension for the case where the divergent velocity condition is applied at 20 km depth. This model has a resolution of 1,024 by 1,024 elements (2.7 km laterally by 0.7 km in depth).

Numerical modeling of mantle flow and temperature at the ridge predicts progressive thickening of the thermally defined plate with distance from the ridge (Figure 7). Translating these temperatures to seismic velocity results in increasing velocities with distance from the ridge, as well as an increasing thickness to the seismically fast lid. Predicted receiver functions include a Moho. However, no discernible LAB phase is predicted owing to the gradual nature of the velocity gradient at the base of the thermally defined plate. A negative feature, follows just beneath the Moho, as a sidelobe artifact. The increase in Moho amplitude away from the ridge axis occurs because the lithosphere is progressively faster.

## 4 Discussion

The shallow positive phase at 3–9 km agrees well with the strong shallow velocity gradients from 0 to 8–10 km in ambient noise tomography and interpreted as the transition from fractured extrusive igneous rocks typically associated with layer two of the oceanic Moho to the deeper intrusive crust (Green et al., 2017). The deeper positive phase at 25–40 km depth likely represents the Moho. In the southern part of the North Iceland Rift Zone (NIRZ) where only a single crustal phase is imaged at intermediate depths, ∼14 km, crustal structure could be more simple (single layer), or the Moho phase may be shallow, thus interfering with the phase from the base of the extrusives. Alternately the deeper Moho phase is weak or not existent and the extrusive layer is very thick.

Our Moho depths are in general agreement with the range of values reported from Ps (Ps) imaging and surface waves, ∼15–42 km (Darbyshire et al., 2000; Kumar et al., 2007; Li & Detrick, 2006), even if details of the models are all slightly different. The general trend of increasing crustal thickness away from the ridge is in agreement with a similar trend in Ps receiver functions from the northern ridge segment (Darbyshire et al., 2000). Beneath the large Bärdarbunga volcano (longitude = −17.5°, lattitude = 64.6°) our 35 km thick crust agrees with the 32 km thick crust from Ps receiver functions (Kumar et al., 2007). It is also within error of the 40 km thickness from refraction (Darbyshire et al., 1998), and Ps receiver function modeling that suggested a Moho velocity gradient from 30 to 40 km (Darbyshire et al., 2000). Although there is general agreement, variability at individual locations could be owing to complications of Ps from reverberated phases related to the shallower discontinuity at 3–9 km depth and/or lower lateral resolution in Sp in comparison to Ps.

The depth of the negative phase agrees with a previous Sp receiver function study which found a decrease in velocity with depth at ∼80 km depth beneath Iceland (Kumar et al., 2005). It is also in agreement with the location of the gradual drop in velocity from 50 to 100 km depth in surface wave velocity across the region and a fast lid that persists throughout (Li & Detrick, 2006). Our result provides higher lateral resolution (25–50 km) than these previous studies (100–250 km). The modeled velocity contrasts from the multitaper (15 ± 7% and 10 ± 5% drops) are in agreement and in some cases slightly greater than the magnitude of the surface wave drops (∼9% beneath the island and ∼5% near the ridge axis). Larger receiver function results are likely explained by lateral or vertical smoothing in the surface waves.

The imaged negative phase is not consistent with predictions for a purely thermal model. Although a negative velocity gradient is predicted beneath the seismically fast lithospheric lid (Figure 7b), the velocity gradient is too gradual, and no strong receiver function phase is predicted (Figure 7c). Particularly beneath the ridge, the seismically fast lithospheric lid is nearly nonexistent and the Moho is weak (Figure 7b). In addition, the depth of the observed negative phase does not correspond to the depth variations of the thermal model. It is deeper than the center of the negative velocity gradient predicted for a thermal model by 20–35 km beneath most the island (Figures 1 and 7b). Its depth variability is similarly more complicated than isotherm patterns for a conductively cooling lithosphere, which predict simple thickening with age (blue phase versus green line, Figure 1). A thermal plume could perturb progressive aging, thinning the lithosphere. However, our areas of thinned lithosphere do not correspond to either the expected plume location (Li & Detrick, 2006) or locations formed during hypothesized periods of hotter plume temperature (Jones et al., 2014). Another mechanism is required to explain the magnitude, sharpness, absolute depth, and lateral depth variations of the imaged velocity drop.

An additional mechanism, such as hydration, melting, or anisotropy can further decrease seismic velocities, in comparison to a thermally defined model. Bulk composition variations cannot explain the magnitude of the seismic velocity drop alone (<2%) (Schutt & Lesher, 2006). An increase in hydration with depth could produce a large velocity reduction if hypothesized grain boundary sliding mechanisms are activated (Karato et al., 2015). Although, a hydrated mantle beneath Iceland is not likely since hydration would partition into the high degrees of mantle melting that is thought to occur beneath Iceland. In addition, recent experimental work suggests that hydration does not impact seismic velocity (Cline et al., 2018). A change in radial anisotropy with depth can only explain a small (< 2%) apparent Sp velocity contrast, far smaller than our observations (Rychert & Harmon, 2017). Alternatively, a small amount of partial melting beneath the discontinuity could easily explain the observed magnitude of the contrast (Clark & Lesher, 2017; Hammond & Humphreys, 2000), and it is suggested that melt naturally ponds in this depth range (Sakamaki et al., 2013). In this case, the observed phase would necessarily represent the LAB, since asthenospheric melt at concentrations imageable by seismic waves (∼1%) (Hammond & Humphreys, 2000) or more (Clark & Lesher, 2017) is expected to significantly weaken the mantle (Hirth & Kohlstedt, 1995; Jackson et al., 2006).

However, to also achieve the observed topography on the discontinuity may require a combination of mechanisms. One possibility is that a compositional boundary dictates the depth at which melt ponds in the mantle. If the overlying lithosphere is compositionally depleted and dehydrated, for instance in clinopyroxene, which occurs at ∼20% depletion, it could strengthen the mantle and create a permeability boundary below which melt ponds (Sparks & Parmentier, 1991). Steady state solid mantle flow and melting would not occur shallower than the discontinuity owing to the strength of the depleted lithosphere and lack of fusible material, which similarly prevents the ascent of melt (Ito et al., 1999). In numerical models, the viscous depleted layer also prevents shallow melting and can produce the relatively modest crustal thickness observed in Iceland (Ito et al., 1999). Steady state numerical models of Iceland predict a viscous layer with a base that varies from 78 to 65 km depth in the spreading direction, in general agreement with our range of observed LAB depths (Ito et al., 1999). The depth variability is smaller than our observations and also with an opposite sense, thicker beneath the ridge. Nonsteady state behavior, like the model proposed here with a pulsing plume could result in greater topography, and provide a better match with our observations. For instance, this boundary would occur at 60 km depth assuming a mantle potential temperature of ∼1,515°C. It would occur deeper during times of increased plume thermal anomalies, for instance ∼84 km for 1,600°C. The depth variability would explain the observed undulations in our negative polarity phase.

The observation of a persistent thick lithosphere beneath the ridge is very different from constraints from nonhot spot affected ridges. For instance, at the fast‐spreading East Pacific Rise surface waves detect no seismically fast lithospheric lid (Harmon et al., 2009). At the intermediate spreading Juan de Fuca and Gorda Ridges receiver functions and surface waves similarly image seismically fast lithosphere that thins toward the ridge axis, reaching <25 km thickness (Audet, 2016; Bell et al., 2016; Rychert et al., 2018). Low seismic velocity and resistivity in these cases are typically interpreted as zones of shallow partial melt (Bell et al., 2016; Harmon et al., 2009; Key et al., 2013; Rychert et al., 2018; Tian et al., 2013).

The observation of a persistent thick lithosphere is similar to previous imaging from hot spot affected ridges. For example, beneath the intermediate‐spreading Nazca Spreading Centre just north of the Galapagos hot spot a deep velocity drop at 40–70 km is imaged with surface waves (Villagomez et al., 2007) and receiver functions (Byrnes et al., 2015; Rychert et al., 2014) across the ridge. These hot spot affected results are typically interpreted as depleted lithospheric material with additional strength (Byrnes et al., 2015; Rychert et al., 2014; Villagomez et al., 2007), lending further support of a compositional lithosphere.

Variations in the thickness of a compositionally depleted layer could be caused by different spreading and upwelling rates or different potential temperatures at the time of formation. Temporal variations in spreading rate are not evidenced here. More likely, episodes of increased thermal plume anomalies explain the observed lithospheric thickness variations. Periods of increased plume temperatures or flux could cause increased melting depths that are reflected in thicker regions of depletion and dehydration, and also thicker tectonic plates (Ito, 2001; Yamamoto & Morgan, 2009). Alternatively, propagation of small scale convective instabilities could periodically increase upwelling (Martinez & Hey, 2017). Indeed, our results show a deep negative phase beneath lithosphere formed during time period of hypothesized high plume temperatures and or flux, 9 Ma (Jones et al., 2002) (Figure 3), with thinner lithosphere in other regions. This suggests that tectonic plate thickness is likely dictated by the conditions of plate formation.

**Figure 8:**
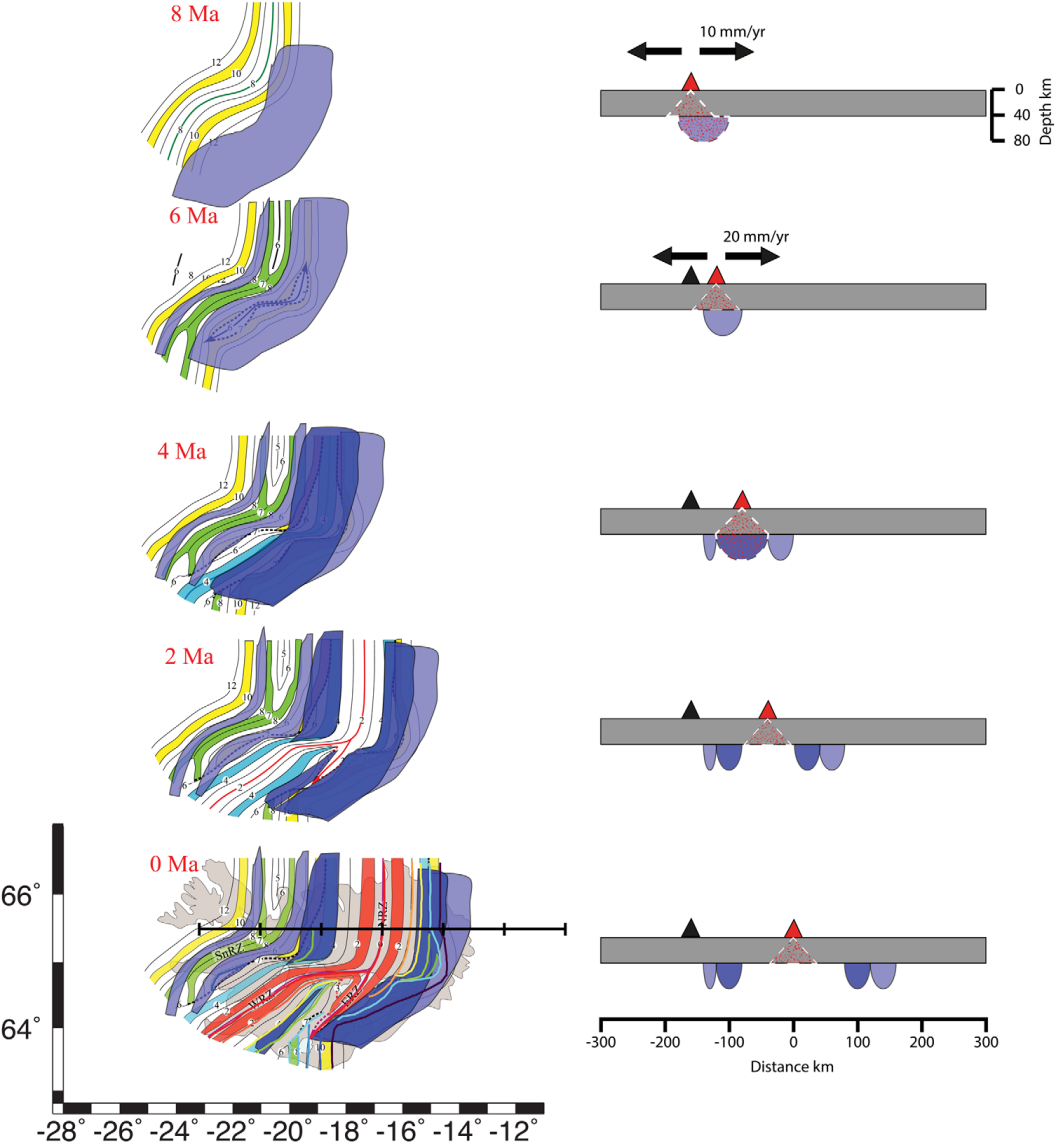
Schematic of the evolution of lithospheric thickness beneath Iceland. The left column shows map views, 8 Ma to present at 2 Ma intervals (top to bottom) with isochons (Ivarsson, 1992). Isochrons are as labeled. Blue areas show hypothesized regions of thickened lithosphere caused by increased plume anomaly at 9 (light blue) and 4 Ma (dark blue). Cross section indicated as thick black line on 0 Ma map. Right column shows cross sections with lithosphere in blue. Triangles show active (red) and extinct (black) spreading ridge. Red hatched regions indicate areas of hypothesized melt generation.

We illustrate a magmatic‐tectonic scenario that could explain the observed variation in lithospheric thickness (Figures 1 and 3) in Figure 8. We assume plume pulsing beneath the ridge at 9 and 4 Ma based on modeling of crustal thickness and geochemical variations at the nearby Reykjanes Ridge (Jones et al., 2014) and use magnetic isochrones and associated spreading rates (Ivarsson, 1992; Martin et al., 2011) to track the formation and evolution of the thickened lithosphere in the region. At 9–8 Ma, a plume pulse created a ∼100 km wide region of thickened lithosphere across the active ridge (Figure 8). The ridge proceeded to spread at 10 mm/yr. At 7–6 Ma, a new ridge formed to the east and also rifted the pre‐existing thickened lithosphere, spreading at 20 mm/yr. The western ridge system died off. At 5–4 Ma, a new plume pulse thickened a ∼100 km wide region beneath the active ridge, creating a nearly continuously thickened lithosphere beneath 4–9 My old lithosphere. From 3 to 0 Ma, the thickened lithosphere rifted apart at 20 mm/yr, thinning the area beneath the ridge. The resulting predicted depth to the LAB from the tectonic model (Figure 8) is strikingly similar to that observed in our result (Figure 3).

Additional positive phases at depth may represent velocity increases related to the reduction of melt concentrations with depth. The positive phase at 80–90 km may represent the base of a highly concentrated melt layer, owing to ponding (Sakamaki et al., 2013). The deeper positive phase near 150 ± 10 km depth agrees with a similar boundary imaged by a previous Sp receiver function result at 135 ± 5 km (Vinnik et al., 2005) and at similar depths beneath other plumes globally (Li et al., 2000; Rychert et al., 2014, 2013). This boundary is likely related to the location of the thermal plume anomaly. This location is just east of the central volcanic region. Possible explanations include mantle plume ascent that is not perfectly vertical (Ballmer et al., 2015; Steinberger & Antretter, 2006; Yamamoto & Morgan, 2009) and/or mantle plume deflection at depths shallower than 150 km (Yamamoto & Morgan, 2009). Indeed, a plume that is located to the east of Iceland at mantle depths is compatible with progressive eastward ridge jumps occurring over the past 14 My (Ivarsson, 1992; Martin et al., 2011). Although the hypothesized location of the thermal plume anomaly is likely outside the well‐resolved area of surface wave inversions, the strongest velocity anomalies in the 75–150 km depth range occur just north or east of Iceland (Li & Detrick, 2006), in agreement with our result.

## References

[ggge21582-bib-0001] Armitage, J. J. , Henstock, T. J. , Minshull, T. A. , & Hopper, J. R. (2008). Modelling the composition of melts formed during continental breakup of the Southeast Greenland margin. Earth and Planetary Science Letters, 269(1–2), 248–258.

[ggge21582-bib-0002] Audet, P. (2016). Receiver functions using OBS data: Promises and limitations from numerical modelling and examples from the Cascadia Initiative. Geophysical Journal International, 205(3), 1740–1755.

[ggge21582-bib-0003] Ballmer, M. D. , Ito, G. , & Cheng, C. (2015). Asymmetric dynamical behavior of thermochemical plumes and implications for Hawaiian Lava composition. Geophysical Monograph Series, 208, 35–57.

[ggge21582-bib-0004] Bell, S. , Ruan, Y. Y. , & Forsyth, D. W. (2016). Ridge asymmetry and deep aqueous alteration at the trench observed from Rayleigh wave tomography of the Juan de Fuca plate. Journal of Geophysical Research, 121, 7298–7321. 10.1002/2016JB012990

[ggge21582-bib-0005] Byrnes, J. S. , Hooft, E. E. E. , Toomey, D. R. , Villagomez, D. R. , Geist, D. J. , & Solomon, S. C. (2015). An upper mantle seismic discontinuity beneath the Galapagos Archipelago and its implications for studies of the lithosphere‐asthenosphere boundary. Geochemistry, Geophysics, Geosystems, 16, 1070–1088. 10.1002/2014GC005694

[ggge21582-bib-0006] Clark, A. N. , & Lesher, C. E. (2017). Elastic properties of silicate melts: Implications for low velocity zones at the lithosphere‐asthenosphere boundary. Science Advances, 3(12), e1701312. 10.1126/sciadv.1701312 PMC5733110 29255800

[ggge21582-bib-0007] Cline, C. J. , Faul, U. H. , David, E. C. , Berry, A. J. , & Jackson, I. (2018). Redox‐influenced seismic properties of uppermantle olivine. Nature, 555(7696), 355. 29542688 10.1038/nature25764

[ggge21582-bib-0008] Darbyshire, F. A. , Bjarnason, I. T. , White, R. S. , & Flovenz, O. G. (1998). Crustal structure above the Iceland mantle plume imaged by the ICEMELT refraction profile. Geophysical Journal International, 135(3), 1131–1149.

[ggge21582-bib-0009] Darbyshire, F. A. , Priestley, K. F. , White, R. S. , Stefansson, R. , Gudmundsson, G. B. , & Jakobsdottir, S. S. (2000). Crustal structure of central and northern Iceland from analysis of teleseismic receiver functions. Geophysical Journal International, 143(1), 163–184.

[ggge21582-bib-0010] Detrick, R. , & Crough, S. (1978). Island subsidence, hot spots, and lithospheric thinning. Journal of Geophysical Research, 83(B3), 1236–1244.

[ggge21582-bib-0011] Fei, H. Z. , Wiedenbeck, M. , Yamazaki, D. , & Katsura, T. (2013). Small effect of water on upper‐mantle rheology based on silicon self‐diffusion coefficients. Nature, 498(7453), 213. 23765497 10.1038/nature12193

[ggge21582-bib-0012] Gaherty, J. B. , Kato, M. , & Jordan, T. H. (1999). Seismological structure of the upper mantle: A regional comparison of seismic layering. Physics of the Earth and Planetary Interiors, 110(1–2), 21–41.

[ggge21582-bib-0013] Green, R. G. , Priestley, K. F. , & White, R. S. (2017). Ambient noise tomography reveals upper crustal structure of Icelandic rifts. Earth and Planetary Science Letters, 466, 20–31.

[ggge21582-bib-0014] Hall, P. S. , & Kincaid, C. (2003). Melting, dehydration, and the dynamics of off‐axis plume‐ridge interaction. Geochemistry, Geophysics, Geosystems, 4(9), 8510. 10.1029/2003GC000567

[ggge21582-bib-0015] Hammond, W. C. , & Humphreys, E. D. (2000). Upper mantle seismic wave velocity: Effects of realistic partial melt geometries. Journal of Geophysical Research, 105(B5), 10975–10986.

[ggge21582-bib-0016] Harmon, N. , Forsyth, D. W. , & Weeraratne, D. S. (2009). Thickening of young Pacific lithosphere from high‐resolution Rayleigh wave tomography: A test of the conductive cooling model. Earth and Planetary Science Letters, 278(1–2), 96–106.

[ggge21582-bib-0017] Helffrich, G. (2006). Extended‐time multitaper frequency domain cross‐correlation receiver‐function estimation. Bulletin of the Seismological Society of America, 96(1), 344–347.

[ggge21582-bib-0018] Hirth, G. , & Kohlstedt, D. L. (1995). Experimental constraints on the dynamics of the partially molten upper‐mantle. 2. Deformation in the dislocation creep regime. Journal of Geophysical Research, 100(B8), 15441–15449.

[ggge21582-bib-0019] Hirth, G. , & Kohlstedt, D. L. (1996). Water in the oceanic upper mantle: Implications for rheology, melt extraction and the evolution of the lithosphere. Earth and Planetary Science Letters, 144(1–2), 93–108.

[ggge21582-bib-0020] Ito, G. (2001). Reykjanes ‘V’‐shaped ridges originating from a pulsing and dehydrating mantle plume. Nature, 411(6838), 681–684. 11395767 10.1038/35079561

[ggge21582-bib-0021] Ito, G. , Shen, Y. , Hirth, G. , & Wolfe, C. J. (1999). Mantle flow, melting, and dehydration of the Iceland mantle plume. Earth and Planetary Science Letters, 165(1), 81–96.

[ggge21582-bib-0022] Ivarsson, G. (1992). *Geology and petrochemistry of the Torfajokull central volcano in Central South Iceland, in association with the Icelandic Hot Spot and Rift Zones* (PhD dissertation, 332 p.). Honolulu, HI: University of Hawaii.

[ggge21582-bib-0023] Jackson, I. , & Faul, U. H. (2010). Grainsize‐sensitive viscoelastic relaxation in olivine: Towards a robust laboratory‐based model for seismological application. Physics of the Earth and Planetary Interiors, 183(1–2), 151–163.

[ggge21582-bib-0024] Jackson, I. , Faul, U. H. , Fitz Gerald, J. D. , & Morris, S. J. S. (2006). Contrasting viscoelastic behavior of melt‐free and melt‐bearing olivine: Implications for the nature of grain‐boundary sliding. Materials Science and Engineering A, 442(1–2), 170–174.

[ggge21582-bib-0025] Jones, S. M. , Murton, B. J. , Fitton, J. G. , White, N. J. , Maclennan, J. , & Walters, R. L. (2014). A joint geochemical‐geophysical record of time‐dependent mantle convection south of Iceland. Earth and Planetary Science Letters, 386, 86–97.

[ggge21582-bib-0026] Jones, S. M. , White, N. , & Maclennan, J. (2002). V‐shaped ridges around Iceland: Implications for spatial and temporal patterns of mantle convection. Geochemistry, Geophysics, Geosystems, 3(10), 1059. 10.1029/2002GC000361

[ggge21582-bib-0027] Jordan, T. H. (1978). Composition and development of continental tectosphere. Nature, 274(5671), 544–548.

[ggge21582-bib-0028] Karato, S. I. , Olugboji, T. , & Park, J. (2015). Mechanisms and geologic significance of the mid‐lithosphere discontinuity in the continents. Nature Geoscience, 8(7), 509–514.

[ggge21582-bib-0029] Kawakatsu, H. , Kumar, P. , Takei, Y. , Shinohara, M. , Kanazawa, T. , Araki, E. , et al. (2009). Seismic evidence for sharp lithosphere‐asthenosphere boundaries of oceanic plates. Science, 324(5926), 499–502. 19390042 10.1126/science.1169499

[ggge21582-bib-0030] Keith, C. M. , & Crampin, S. (1977). Seismic body waves in anisotropic media—Synthetic seismograms. Geophysical Journal of the Royal Astronomical Society, 49(1), 225–243.

[ggge21582-bib-0031] Kelemen, P. B. , Hirth, G. , Shimizu, N. , Spiegelman, M. , & Dick, H. J. B. (1997). A review of melt migration processes in the adiabatically upwelling mantle beneath oceanic spreading ridges. Philosophical Transactions of the Royal Society A, 355(1723), 283–318.

[ggge21582-bib-0032] Key, K. , Constable, S. , Liu, L. , & Pommier, A. (2013). Electrical image of passive mantel upwelling beneath the northern east Pacific Rise. Nature, 495(7442), 499–502. 23538832 10.1038/nature11932

[ggge21582-bib-0033] Korenaga, J. , & Karato, S. I. (2008). A new analysis of experimental data on olivine rheology. Journal of Geophysical Research, 113, B02403. 10.1029/2007JB005100

[ggge21582-bib-0034] Kumar, P. , Kind, R. , Hanka, W. , Wylegalla, K. , Reigber, C. , Yuan, X. , et al. (2005). The lithosphere‐asthenosphere boundary in the North‐West Atlantic region. Earth and Planetary Science Letters, 236(1–2), 249–257.

[ggge21582-bib-0035] Kumar, P. , Kind, R. , Priestley, K. , & Dahl‐Jensen, T. (2007). Crustal structure of Iceland and Greenland from receiver function studies. Journal of Geophysical Research, 112, B03301. 10.1029/2005JB003991

[ggge21582-bib-0036] Levy, F. , & Jaupart, C. (2011). Temperature and rheological properties of the mantle beneath the North American craton from an analysis of heat flux and seismic data. Journal of Geophysical Research, 116, B01408. 10.1029/2010JB007726

[ggge21582-bib-0037] Li, A. B. , & Burke, K. (2006). Upper mantle structure of southern Africa from Rayleigh wave tomography. Journal of Geophysical Research, 111, B10303. https://doi.org/10310.11029/12006JB004321

[ggge21582-bib-0038] Li, A. B. , & Detrick, R. S. (2006). Seismic structure of Iceland from Rayleigh wave inversions and geodynamic implications. Earth and Planetary Science Letters, 241(3–4), 901–912.

[ggge21582-bib-0039] Li, X. , Kind, R. , Priestley, K. , Sobolev, S. V. , Tilmann, F. , Yuan, X. , et al. (2000). Mapping the Hawaiian plume conduit with converted seismic waves. Nature, 405(6789), 938–941. 10879532 10.1038/35016054

[ggge21582-bib-0040] Li, X. Q. , Kind, R. , Yuan, X. H. , Wolbern, I. , & Hanka, W. (2004). Rejuvenation of the lithosphere by the Hawaiian plume. Nature, 427(6977), 827–829. 14985758 10.1038/nature02349

[ggge21582-bib-0041] Martin, E. , Paquette, J. L. , Bosse, V. , Ruffet, G. , Tiepolo, M. , & Sigmarsson, O. (2011). Geodynamics of rift‐plume interaction in Iceland as constrained by new Ar‐40/Ar‐39 and in situ U‐Pb zircon ages. Earth and Planetary Science Letters, 311(1–2), 28–38.

[ggge21582-bib-0042] Martinez, F. , & Hey, R. (2017). Propagating buoyant mantle upwelling on the Reykjanes Ridge. Earth and Planetary Science Letters, 457, 10–22.

[ggge21582-bib-0043] Moresi, L. , Zhong, S. J. , & Gurnis, M. (1996). The accuracy of finite element solutions of Stokes' flow with strongly varying viscosity. Physics of the Earth and Planetary Interiors, 97(1–4), 83–94.

[ggge21582-bib-0044] Nielsen, T. K. , & Hopper, J. R. (2004). From rift to drift: Mantle melting during continental breakup. Geochemistry, Geophysics, Geosystems, 5, Q07003. 10.1029/2003GC000662

[ggge21582-bib-0045] Parsons, B. , & Sclater, J. G. (1977). Analysis of variation of ocean‐floor bathymetry and heat‐flow with age. Journal of Geophysical Research, 82(5), 803–827.

[ggge21582-bib-0046] Rychert, C. A. , Fischer, K. M. , & Rondenay, S. (2005). A sharp lithosphere‐asthenosphere boundary imaged beneath eastern North America. Nature, 436(7050), 542–545. 16049485 10.1038/nature03904

[ggge21582-bib-0047] Rychert, C. A. , & Harmon, N. (2017). Constraints on the anisotropic contributions to velocity discontinuities at ∼60 km depth beneath the Pacific. Geochemistry, Geophysics, Geosystems, 18, 2855. 10.1002/2017GC006850 PMC5652234 29097907

[ggge21582-bib-0048] Rychert, C. A. , Harmon, N. , & Ebinger, C. (2014). Receiver function imaging of lithospheric structure and the onset of melting beneath the Galapagos Archipelago. Earth and Planetary Science Letters, 388, 156–165.

[ggge21582-bib-0049] Rychert, C. A. , Harmon, N. , & Tharimena, S. (2018). Scattered wave imaging of the oceanic plate in Cascadia. Science Advances, 4(2), eaao1908. 10.1126/sciadv.aao1908 PMC5812736 29457132

[ggge21582-bib-0050] Rychert, C. A. , Hammond, J. O. S. , Harmon, N. , Kendall, J. M. , Keir, D. , Ebinger, C. , et al. (2012). Volcanism in the Afar Rift sustained by decompression melting with minimal plume influence. Nature Geoscience, 5(6), 406–409.

[ggge21582-bib-0051] Rychert, C. A. , Laske, G. , Harmon, N. , & Shearer, P. M. (2013). Seismic imaging of melting in a displaced Hawaiian Plume. Nature Geoscience, 6(8), 657–660.

[ggge21582-bib-0052] Rychert, C. A. , Rondenay, S. , & Fischer, K. M. (2007). P‐to‐S and S‐to‐P imaging of a sharp lithosphere‐asthenosphere boundary beneath eastern North America. Journal of Geophysical Research, 112, B08314. 10.1029/2006JB004619 16049485

[ggge21582-bib-0053] Rychert, C. A. , & Shearer, P. M. (2009). A global view of the lithosphere‐asthenosphere boundary. Science, 324(5926), 495–498. 19390041 10.1126/science.1169754

[ggge21582-bib-2254] Rychert, C. A., & Harmon, N. (2018). Predictions and observations for the oceanic lithosphere from S‐to‐P receiver functions and SS precursors. *Geophysical Research Letters*, *45* 10.1029/2018GL077675 PMC6049891 30034045

[ggge21582-bib-0054] Rychert, C. A. , Shearer, P. M. , & Fischer, K. M. (2010). Scattered wave imaging of the lithosphere‐asthenosphere boundary. Lithos, 120(1–2), 173–185.

[ggge21582-bib-0055] Sakamaki, T. , Suzuki, A. , Ohtani, E. , Terasaki, H. , Urakawa, S. , Katayama, Y. , et al. (2013). Ponded melt at the boundary between the lithosphere and asthenosphere. Nature Geoscience, 6(12), 1041–1044.

[ggge21582-bib-0056] Schutt, D. L. , & Lesher, C. E. (2006). Effects of melt depletion on the density and seismic velocity of garnet and spinel lherzolite. Journal of Geophysical Research, 111, B05401. 10.1029/2003JB002950

[ggge21582-bib-0057] Shearer, P. M. , & Orcutt, J. A. (1987). Surface and near‐surface effects on seismic‐waves‐theory and borehole seismometer results. Bulletin of the Seismological Society of America, 77(4), 1168–1196.

[ggge21582-bib-0058] Sparks, D. , & Parmentier, E. (1991). Melt extraction from the mantle beneath spreading centers. Earth and Planetary Science Letters, 105(4), 368–377.

[ggge21582-bib-0059] Staples, R. K. , White, R. S. , Brandsdottir, B. , Menke, W. , Maguire, P. K. H. , & McBride, J. H. (1997). Faroe‐Iceland ridge experiment. 1. Crustal structure of northeastern Iceland. Journal of Geophysical Research, 102(B4), 7849–7866.

[ggge21582-bib-0060] Steinberger, B. , & Antretter, M. (2006). Conduit diameter and buoyant rising speed of mantle plumes: Implications for the motion of hot spots and shape of plume conduits. Geochemistry, Geophysics, Geosystems, 7, Q11018. 10.1029/2006GC001409

[ggge21582-bib-0061] Tan, Y. , & Helmberger, D. V. (2007). Trans‐Pacific upper mantle shear velocity structure. Journal of Geophysical Research, 112, B08301. 10.1029/2006JB004853

[ggge21582-bib-0062] Tharimena, S. , Rychert, C. , Harmon, N. , & White, P. (2017). Imaging Pacific lithosphere seismic discontinuities: Insights from SS precursor modeling. Journal of Geophysical Research: Solid Earth, 122, 2131–2152. 10.1002/2016JB013526

[ggge21582-bib-0063] Tian, Y. , Shen, W. S. , & Ritzwoller, M. H. (2013). Crustal and uppermost mantle shear velocity structure adjacent to the Juan de Fuca Ridge from ambient seismic noise. Geochemistry, Geophysics, Geosystems, 14, 3221–3233. 10.1002/ggge.20206

[ggge21582-bib-0064] Tryggvason, E. , & Bath, M. (1961). Upper crustal structure of Iceland. Journal of Geophysical Research, 66(6), 1913–1925.

[ggge21582-bib-0065] Venzke, E. e. (2013). Volcanoes of the world (Vol. 4.6.7). Washington, DC: Smithsonian Institution.

[ggge21582-bib-0066] Villagomez, D. R. , Toomey, D. R. , Hooft, E. E. E. , & Solomon, S. C. (2007). Upper mantle structure beneath the Galapagos Archipelago from surface wave tomography. Journal of Geophysical Research, 112, B07303. 10.1029/2006JB004672

[ggge21582-bib-0067] Vinnik, L. P. , Foulger, G. R. , & Du, Z. (2005). Seismic boundaries in the mantle beneath Iceland: A new constraint on temperature. Geophysical Journal International, 160(2), 533–538.

[ggge21582-bib-0068] Weir, N. R. W. , White, R. S. , Brandsdottir, B. , Einarsson, P. , Shimamura, H. , Shiobara, H. , et al. (2001). Crustal structure of the northern Reykjanes Ridge and Reykjanes Peninsula, southwest Iceland. Journal of Geophysical Research, 106(B4), 6347–6368.

[ggge21582-bib-0069] Yamamoto, M. , & Morgan, J. P. (2009). North Arch volcanic fields near Hawaii are evidence favouring the restite‐root hypothesis for the origin of hotspot swells. Terra Nova, 21(6), 452–466.

